# Prediction model and assessment of malnutrition in patients with stable chronic obstructive pulmonary disease

**DOI:** 10.1038/s41598-024-56747-2

**Published:** 2024-03-18

**Authors:** Xurui Shen, Ruiqi Qian, Yuan Wei, Zhichao Tang, Huafei Zhong, Jianan Huang, Xiuqin Zhang

**Affiliations:** 1 Department of Respiratory Medicine, The First Affiliated Hospital of Soochow University, Suzhou, Jiangsu Province China; 2 Dushu Lake Hospital Affiliated to Soochow University, Suzhou, China

**Keywords:** Nutritional status, Body composition, Fat-free mass index, Computational biology and bioinformatics, Respiratory tract diseases

## Abstract

Chronic obstructive pulmonary disease (COPD) combined with malnutrition results in decreased exercise capacity and a worse quality of life. We aimed to develop an observational case–control study to explore the effective and convenient method to identify potential individuals is lacking. This study included data from 251 patients with COPD and 85 participants in the control group. Parameters and body composition were compared between groups, and among patients with varied severity. The LASSO approach was employed to select the features for fitting a logistic model to predict the risk of malnutrition in patients with stable COPD. Patients with COPD exhibited significantly lower 6-min walk distance (6MWD), handgrip strength, fat-free mass index (FFMI), skeletal muscle mass (SMM) and protein. The significant predictors identified following LASSO selection included 6MWD, waist-to-hip ratio (WHR), GOLD grades, the COPD Assessment Test (CAT) score, and the prevalence of acute exacerbations. The risk score model yielded good accuracy (C-index, 0.866 [95% CI 0.824–0.909]) and calibration (Brier score = 0.150). After internal validation, the adjusted C-index and Brier score were 0.849, and 0.165, respectively. This model may provide primary physicians with a simple scoring system to identify malnourished patients with COPD and develop appropriate rehabilitation interventions.

## Introduction

Chronic obstructive pulmonary disease (COPD) is a common, preventable and treatable chronic respiratory disease characterized by persistent respiratory symptoms and airflow limitation^1^. Growing evidence suggests that COPD is associated with various extrapulmonary effects, including changes in nutritional status and body composition^2^. Previous studies showed that the incidence of malnutrition in patients with COPD was 30–60%^3,4^, leading to reduced exercise tolerance, higher risk of acute exacerbations and lower quality of life. Previous experts have used body mass index (BMI) less than 20 kg/m^2^ as a diagnostic criterion for malnutrition^5^, however, it is important to note that BMI may not accurately capture the nuanced alterations in an individual's body composition. Therefore, this observational case–control study combined fat-free mass index (FFMI) to assess the nutritional status of stable COPD with the body composition analyzer and explored the correlation between FFMI with pulmonary function indices, exercise capacity and clinical symptoms. To effectively implement early intervention strategies aimed at delaying the progression of deterioration, we also constructed a prediction model for recognizing patients that probably combined with malnutrition.

## Methods

### Study population

Our study recruited 251 male outpatients with stable COPD from October 2019 to December 2021 at the First Affiliated Hospital of Soochow University diagnosed according to the Global Initiative for Chronic Obstructive Lung Disease. Another 85 healthy participants during the same period were also included as the control group. They were randomly recruited by posting flyers on bulletin boards as well as from data collected at the hospital. Informed consent was obtained from all subjects and this study was approved by the research ethics committee of the first hospital affiliated to Soochow university (NO. 2022088).

### Information collection

We collected the demographic and clinical characteristics of all participants, including their age, height, weight, history of smoking, acute exacerbations during the past year, and evaluation of symptoms with the COPD Assessment Test (CAT) and Modified British Medical Research Council (mMRC).

All the enrolled patients underwent spirometry using a Master screen-PFT system (Vyaire, Mettawa, Ill, USA) in the Pulmonary Function Test Room, and the forced expiratory volume during one second (FEV1) and forced vital capacity (FVC) were measured, according to the American Thoracic Society (ATS) consensus guidelines^6^. The FEV1, FEV1/FVC, and the ratio of FEV1 to predicted FEV1(FEV1%) after inhaling bronchodilators were recorded. According to the category of airflow limitation in COPD (based on FEV1 after the administration of a bronchodilator) in patients with FEV1/FVC < 0.70, stable COPD was divided into four GOLD grades: mild (GOLD 1), ≥ 80% of predicted FEV1; moderate (GOLD 2), 50–80% of predicted FEV1; severe (GOLD 3), 30–50% of predicted FEV1; very severe (GOLD 4), < 30% of predicted FEV1. 6-min walk test was performed according to guideline at the time of inclusion by investigator^7^. Meanwhile, the participants' handgrip strength was measured with a WCS-100 electronic handgrip dynamometer (produced by Shanghai Yilian Science and Education Equipment Co., LTD.), with the maximum value of the dominant hand recorded.

Body composition was assessed using a BCA-2A bioelectrical impedance body composition analyzer (Tsinghua Tongfang Co., Ltd., Beijing, China) by specialized technician in the mean time. Body weight, waist-to-hip ratio (WHR), fat-free mass (FFM), fat mass (FM), muscle mass, skeletal muscle mass (SMM), visceral fat area (VFA) and other body composite variables were measured. The FFMI was calculated as the FFM/(height)^2^. According to the European Society for Clinical Nutrition and Metabolism (ESPEN)^8^, individuals with a BMI below 20 kg/m^2^ if they are under 70 years of age, or below 22 kg/m^2^ if they are 70 years of age or older, or a FFMI below 17 kg/m^2^ in males, were classified as having malnutrition.

### Variables selection and construction of nomogram

We developed the risk-prediction model for the occurrence of malnutrition in stable COPD patients following the transparent reporting of a multivariable-prediction model for Individual Prognosis or Diagnosis guidelines^9^. The variables included patients’ age, handgrip strength, 6-min walk distance (6MWD), WHR, acute exacerbations during the past year, the history of smoking, CAT scores, FVC and FEV1%. We log-transformed values of WHR that was not normally distributed for inclusion in the model. 6MWD was converted into categorical variables, and the roc curve was used to determine the optimal cut-off. Besides, we used a restricted cubic spline to flexibly model and verify the linear relationship between continuous variable and outcome. After transforming, the least absolute shrinkage and selection operator (LASSO) method was taken for variable selection in the training set. In the LASSO approach, the value of λ is used to penalize the absolute magnitude of a regression model's coefficients. The estimates approach zero more as the penalty is increased. As a result, irrelevant variables have zero coefficients, which can be used to eliminate non-influential predictors from the final model^10^.

The model was developed based on the selected features and refitted to avoid model overfitting. A nomogram was then constructed by using a linear combination of the selected features weighted by their regression coefficients. "Points" indicates the score of the corresponding factor below and "Total Points" indicates the summation of all the scores of factors above.

The discriminative ability of the nomogram was evaluated by using Harrell's concordance index (C-index), which was analogous to the area under the receiver operating characteristic curve (AUC). Further, a calibration curve was plotted and the Brier score was calculated to evaluate the agreement between the predicted and actual risk. When the Brier score ≤ 0.25, the model was considered to have favorable calibration. Internal validity and adjustment for overfitting of the nomogram were implemented with a bootstrap resampling (100 times) analysis.

### Statistical analysis

Continuous variables were expressed as mean standard deviation or median interquartile range, while categorical values were expressed using relative frequencies and proportions. Comparisons of parameters between 2 different groups were conducted with the t-test and the Mann–Whitney U test for continuous variables with or without normal distribution. Categorical variables were evaluated using the chi-square test or Fischer exact test. Data were analyzed using SPSS software (Version 24.0), and R software (Version 4.1.0). A *p*-value < 0.05 was considered statistically significant.

## Results

This study included 251 male stable COPD cases in the COPD group and 85 cases in the control group. As shown in Table 1, COPD patients presented significantly lower BMI, 6MWD, handgrip strength, FFMI, SMM, protein, and inorganic salt and mostly had a smoking habit (Table 1).

**Table 1:** Demographic and clinical characteristics of study participants.

Group	Stable COPD (n = 251)	Control (n = 85)	t/Z/χ^2^ value	*P* value
Age (years)	64.84 ± 8.33	57.25 ± 9.14	7.087	** < 0.001**
Height (cm)	168.06 ± 6.33	168.65 ± 5.65	-0.761	0.447
Smoking	224 (89.24%)	53 (62.35%)	31.716	** < 0.001**
BMI (kg/m^2^)	23.33 ± 3.06	24.32 ± 2.69	-2.825	**0.005**
6MWD (m)	530 (490,570)	600 (570,620)		** < 0.001**
Handgrip (kg)	35.48 ± 7.40	36.91 ± 4.18	2.196	**0.029**
IC (L)	2.49 ± 0.66	2.92 ± 0.62	-5.230	** < 0.001**
FVC (L)	3.47 ± 0.88	3.95 ± 0.66	-5.323	** < 0.001**
FEV1 (L)	1.90 ± 0.81	3.05 ± 0.52	-15.273	** < 0.001**
FEV1% (%)	65.47 ± 24.04	98.36 ± 11.73	-16.610	** < 0.001**
FEV1/FVC (%)	53.00 ± 13.84	77.32 ± 4.45	-24.364	** < 0.001**
FFMI (kg/m^2^)	17.34 ± 1.60	18.35 ± 1.31	5.250	** < 0.001**
SMM (kg)	26.92 ± 3.86	29.02 ± 3.35	4.470	** < 0.001**
Protein (kg)	9.58 ± 1.28	10.29 ± 1.12	4.535	** < 0.001**
Inorganic salt (kg)	3.24 ± 0.44	3.43 ± 0.41	3.421	**0.001**

*BMI* body mass index, *6MWD* 6-min walk distance, *IC* inspiratory capacity, *FVC* forced vital capacity, *FEV1* forcedexpiredvolumein1sec, *FFMI* fat-free mass index, *SMM* skeletal muscle mass.

Significant values are in bold.

There were significant differences in age, weight, AE during the past year, CAT, mMRC, BMI, and skeletal muscle mass among different COPD grades when COPD patients were divided into four subgroups based on the FEV1% (Table 2). Compared with Grade I and II patients, Grade III and IV patients showed lower handgrip and 6MWD. The incidence of malnutrition corresponding to grades I, II, III, and IV was 30.86%, 36.36%, 63.49%, and 73.68% respectively.

**Table 2:** Comparison of clinical symptoms, nutritional status, exercise endurance, grip strength and malnutrition rate among different COPD grades.

	Grade I	Grade II	Grade III	Grade IV	*P* value
N	81	88	63	19	
Age (years)	62.48 ± 8.95^b^	65.09 ± 8.58^ab^	66.65 ± 7.04^a^	67.74 ± 5.90^a^	**0.007**
Height (cm)	168.41 ± 6.50	168.31 ± 6.50	167.52 ± 6.26	167.26 ± 5.26	0.772
Handgrip (kg)	40.54 ± 6.78^a^	35.64 ± 6.88^b^	31.93 ± 5.32^c^	27.18 ± 6.28^d^	** < 0.001**
6MWD (m)	572.79 ± 47.61^a^	528.18 ± 51.22^b^	488.91 ± 78.16^c^	368.32 ± 74.76^d^	** < 0.001**
AE (times)	0 (0,1)^d^	1 (0,2)^c^	2 (1,3)^b^	4 (4,6)^a^	** < 0.001**
CAT	5 (3,9)^c^	9 (5,13)^b^	17 (12,20)^a^	20 (17,24)^a^	** < 0.001**
mMRC	0 (0,1)^c^	1 (0,2)^b^	2 (2,3)^a^	3 (3,3)^a^	** < 0.001**
BMI (kg/m^2^)	23.82 ± 2.87^ab^	23.89 ± 2.80^a^	22.62 ± 3.17^bc^	21.04 ± 3.27^c^	** < 0.001**
VFA > 100cm^2^	14 (17.28%)	15 (17.05%)	15 (23.81%)	3 (15.79%)	0.809
WHR	0.87 (0.84,0.92)	0.85 (0.83,0.92)	0.88 (0.85,0.90)	0.88 (0.85,0.93)	0.395
SMM (kg)	28.08 ± 3.90^a^	27.57 ± 3.62^a^	25.49 ± 3.30^b^	23.73 ± 3.56^b^	** < 0.001**
FFMI (kg/m^2^)	17.93 ± 1.45^a^	17.65 ± 1.41^a^	16.64 ± 1.42^b^	15.64 ± 1.69^c^	** < 0.001**
Malnutrition	25 (30.86%)^b^	32 (36.36%)^b^	40 (63.49%)^a^	14 (73.68%)^a^	** < 0.001**

*6MWD* 6-min walk distance, *AE* acute exacerbations during the past year, *BMI* body mass index, *VFA* visceral fat area, *WHR* waist-to-hip ratio, *FFMI* fat-free mass index, *SMM* skeletal muscle mass.

*The significant differences between different grades were compared and shown by letter marking method. Data exhibiting significant differences (p < 0.05) are denoted by different letters.

Significant values are in bold.

All clinical variables were included in the LASSO method selection to construct a prediction model with our cohort. The most regularized and parsimonious model with a tuning λ (log scale) giving a cross-validated error within one standard error of the minimum included 5 significant variables. The results of multivariable logistic analysis were shown in Table 3.

**Table 3:** Multivariate logistic analysis of predictors related to the comorbidity of malnutrition in patients with stable COPD (N = 251).

	β	S.E	z value	P value	OR (95%CI)
6MWD > 567m	− 1.749	0.489	− 3.575	0.000	0.174 (0.063 ~ 0.437)
lg (WHR)	− 20.902	6.032	− 3.465	0.000	0.000 (0.000 ~ 0.000)
GOLD2	− 0.933	0.445	− 2.096	0.036	0.394 (0.161 ~ 0.927)
GOLD3	− 1.163	0.562	− 2.070	0.038	0.313 (0.100 ~ 0.915)
GOLD4	− 2.323	0.877	− 2.648	0.008	0.098 (0.017 ~ 0.545)
AE > 2	1.709	0.500	3.441	0.001	5.523 (2.160 ~ 15.409)
CAT (11–20)	1.795	0.406	4.416	0.000	6.018 (2.765 ~ 13.713)
CAT (> 20)	3.205	0.960	3.337	0.001	24.644 (4.290 ~ 209.404)
Intercept	− 1.615	0.507	− 3.186	0.001	0.199 (0.071 ~ 0.525)

*6MWD* 6-min walk distance, *WHR* waist-to-hip ratio, *AE* acute exacerbations during the past year.

**Figure 1:**
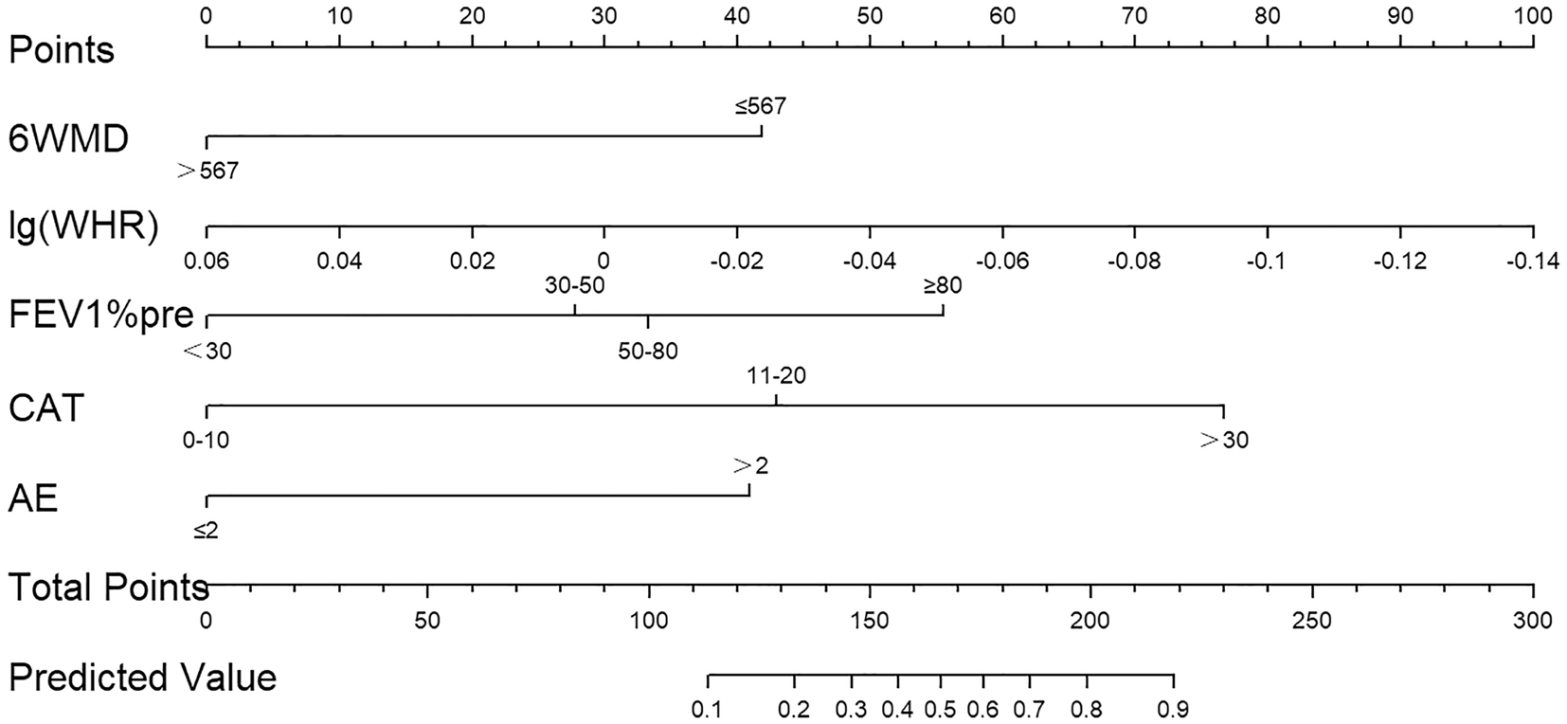
Nomogram for predicting risk of malnutrition for stable COPD patients. Values for each variable are individually plotted and correspond to point values assigned from the point scale (top). These point values are then totaled and plotted on the total point scale (bottom), which is used to assign a corresponding value for prediction. *6MWD* 6-min walk distance, *WHR* waist-to-hip ratio, *AE* acute exacerbations during the past year.

A nomogram was constructed using the results to estimate the risk of malnutrition for patients with COPD (Fig. 1). Clinical scores were assigned to the 5 independent factors and the estimated risk of progression was calculated by summing the scores of each factor. The final score ranged from a minimum of zero points to a maximum of 300 points, where a straight line is drawn to determine the probability of response. This score-based nomogram allowed for a manual estimation of the cumulative risk of malnutrition for each patient. For example, a stable COPD patient with waist-hip ratio measured as 0.88 and finished the 6MWD test at 450 m. He underwent twice exacerbations over the last year, his baseline FEV1% was 45.3% with CAT score evaluated as 18. The total score was about 167, indicating that his risk of malnutrition was approximately 50%.

**Figure 2:**
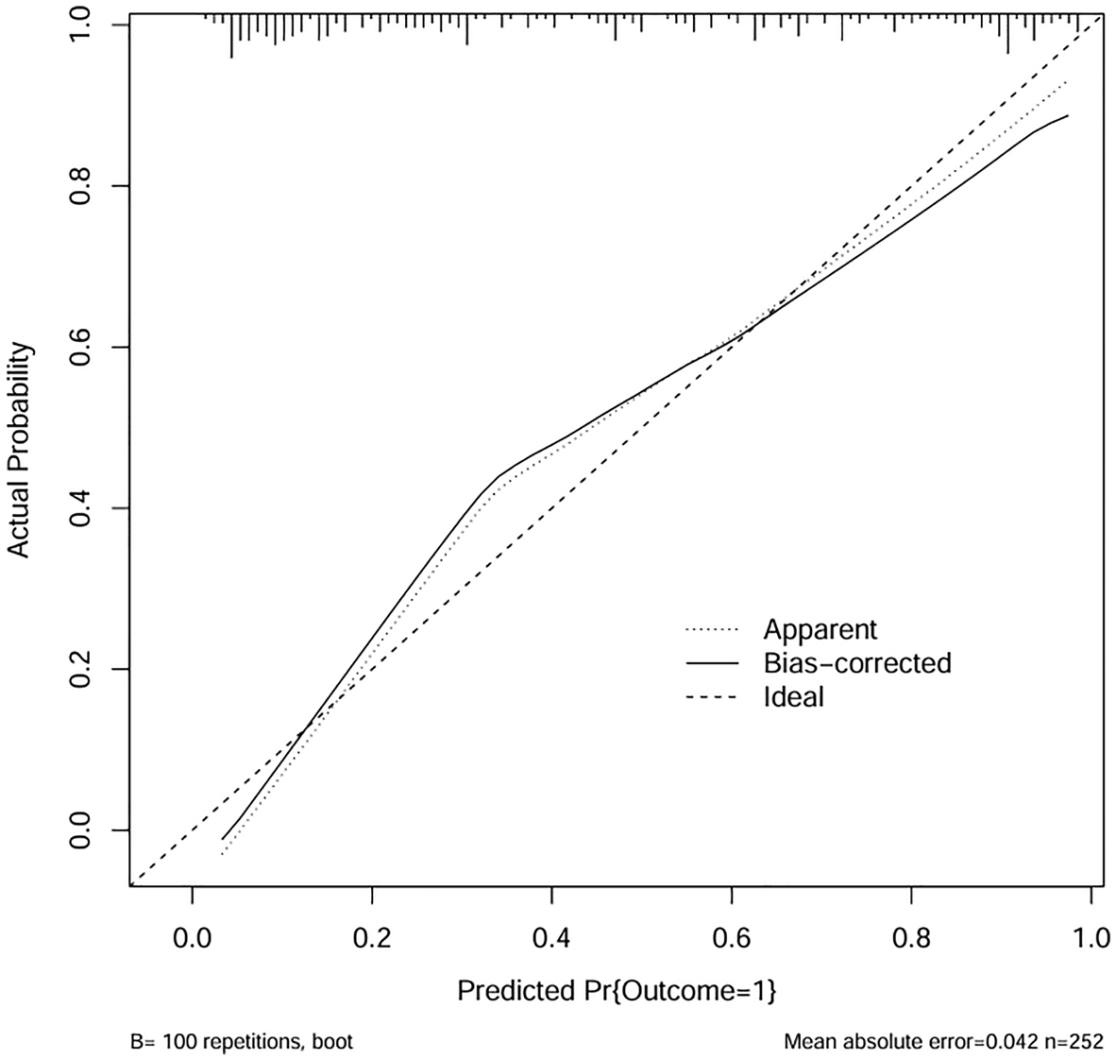
The calibration plot after bootstrap validation.

**Figure 3:**
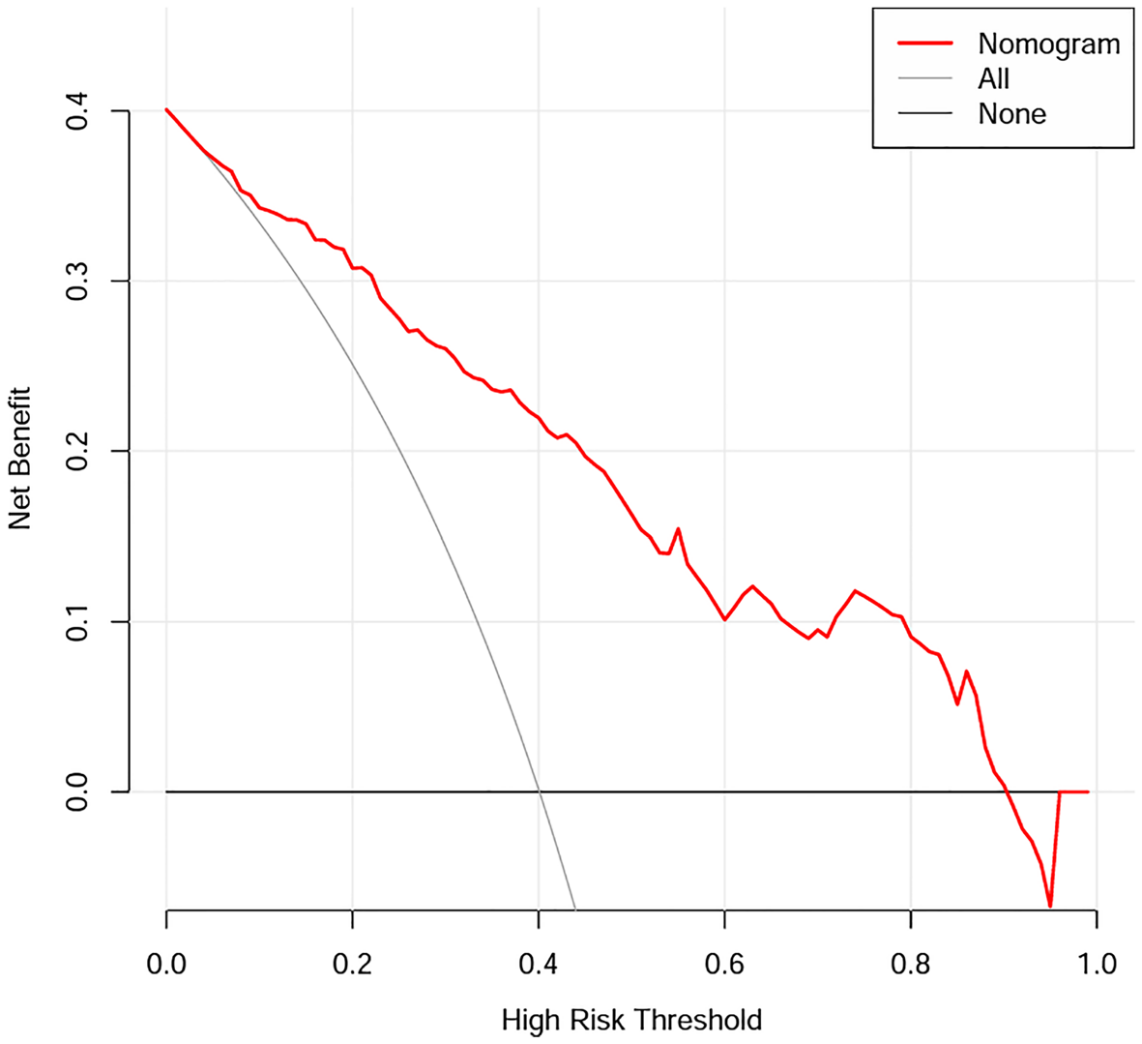
Decision curve analysis of the model.

**Figure 4:**
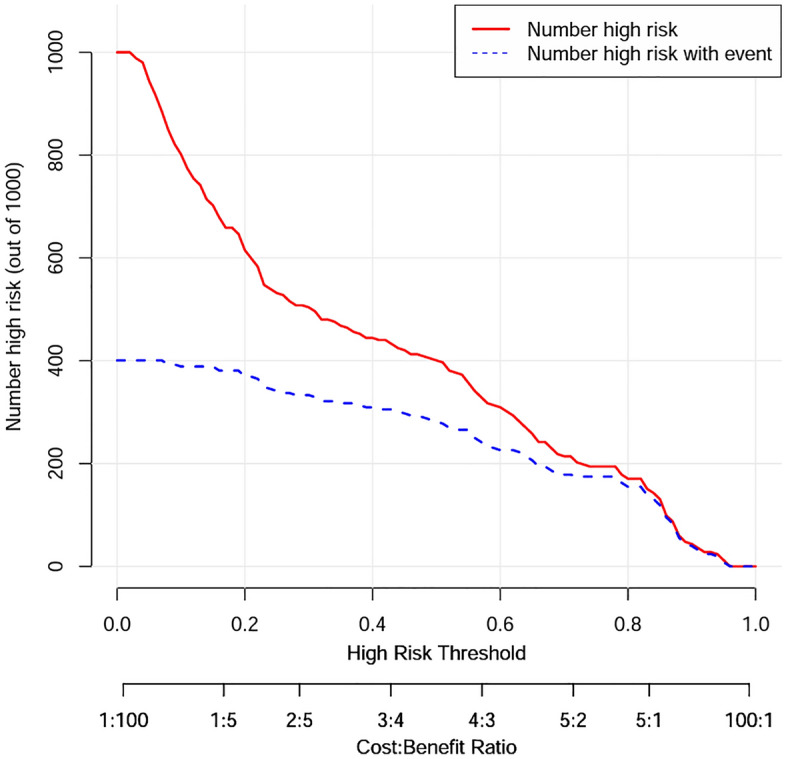
The clinical impact curve of the model.

Our model presented very good discrimination (C-index, 0.866 [95% CI 0.824–0.909]) and calibration (Brier score = 0.150). After bootstrap validation (100 repetitions), the corrected C-index and Brier score were 0.849, and 0.165, respectively. The calibration curve (Fig. 2) showed good agreement between actual and predicted probabilities, and a bootstrap procedure (100 repetitions) provided a bias-corrected estimate of the calibration curve (mean absolute error = 0.042). The DCA curve utilized to evaluate the clinical utility of the nomogram is plotted in Fig. 3. It showed that intervening on patients on the basis of the prediction model leads to higher benefit than the alternative strategies of treating all patients. To evaluate the clinical effects of the nomogram model more visually, the CIC curve (Fig. 4) on the ground of DCA curve was drawn. The “Number high risk” curve was closely to the “Number high risk with event” curve, which indicated that the nomogram model owns extraordinary predictive power.

## Discussion

The results of this study suggested that malnutrition was more common in COPD patients than in controls and got worse with the aggravation of airflow restriction. We enrolled 251 stable COPD patients to investigate the factors correlated with malnutrition. 6MWD, WHR, GOLD grades, AE and CAT score were significant predictors of complication of malnutrition after multivariable analysis. Finally, a nomogram was constructed to visually predict the risk of malnutrition in COPD patients with the basic clinical characteristics.

As shown in our study, the overall proportion of malnutrition in patients with stable COPD is 44.2%, which is in accordance with 30–60% worldwide^3,4^. 6-min walking test and grip strength are experiments used to roughly evaluate the exercise capacity of COPD patients in community practice. Previous studies^11^ showed that 6MWD was associated with pulmonary function parameters including FEV1% pred, FEV1/FVC, PEF and VC in patients with severe and very severe COPD. This study showed that the stable COPD patients’ activity tolerance was significantly lower than that of the control group. Furthermore, there was a substantial difference in skeletal muscle atrophy between COPD patients and healthy controls, aligning with the findings of van de Bool et al.^12^ that the proportion of muscle fibers were decreased in patients with COPD. Several reasons may account for the decline in skeletal muscle occurring in patients with COPD. The primary cause of rapid muscle mass and strength decline is an excessive breakdown of protein, often accompanied by a decrease in protein synthesis, as demonstrated in our study^13^. The systemic inflammatory response of COPD patients leads to peripheral muscle dysfunction^14^, with the type of muscle fibers shifted, resulting in worse muscle strength^15^. Furthermore, it has been observed that chronic hypoxia in patients with COPD can activate mediators that leads to a decrease in the oxygen content and a reduction in the capacity for oxygen transport. These physiological changes might subsequently result in alterations to the structure of the muscles^16^. Meanwhile, the dyspnea symptoms limit patients' exercise, which in turn leads to muscle atrophy and decreased exercise capacity^17^.

With the aggravation of COPD severity classified by GOLD grades, the patients' body weight, BMI, FFMI and SMM were all on a decreasing trend. The probability of combined malnutrition in COPD patients is significantly higher in grades III and IV, along with the decrease in exercise capacity. According to a related study, VFA > 100 cm^2^ was used as a threshold value to compare visceral fat area in patients with different stages of COPD and suggested that moderate-to-severe COPD patients had excessive visceral fat^18^. Although the difference was not statistically significant, we also observed the increase in severe COPD patients. This phenomenon may have something to do with the pronounced decline in skeletal muscle mass of the patients but is also related to the abnormal metabolic states and comorbidities of hypertension or diabetes. Malnutrition and excessive body fat^19^ are both harmful to the physical function and risk of cardiovascular disease for elderly patients.

Hence, the recognition and alleviation of muscle atrophy appears as a significant therapeutic target. As community healthcare plays a pivotal role in managing chronic condition while limited by equipment, the identification of malnutrition in patients with COPD holds significant therapeutic value. The strengths of this study include its status as a pioneer in developing and validating the first nomogram based on the Chinese clinical population by using a LASSO logistic model. According to our findings, 6MWD, waist-to-hip ratio, GOLD grades, CAT score and acute exacerbations during the past year were effective in predicting the risk of combined malnutrition. Airflow obstruction in COPD patients was significantly worse in the malnourished group than in the nutritionally normal group^20,21^, which is in line with previous study presented that FFMI has a positive association with FEV1% and FEV1/FVC^22,23^. Exacerbations in COPD patients are associated with decrease in fat-free mass^24^ and accelerated skeletal muscle loss^25^. Increased airflow limitation and exacerbations in patients lead to increased symptoms of dyspnea and higher CAT values, which in turn impair exercise capacity and affect patients’ quality of life^26^. Past studies measured handgrip to judge muscle mass^27^, while the relationship between exercise capacity and malnutrition remains uncertain. Our results revealed that 6MWD and handgrip strength were strongly correlated with skeletal muscle mass and nutritional status.

The predictors also suggested that augmenting the frequency of exercise sessions and improving patients' muscle strength, along with implementing nutritional interventions to alleviate dyspnea symptoms and enhance lung function, can effectively reduce the risk of combined malnutrition in stable COPD patients. Except for taking inhaled medications regularly, pulmonary rehabilitation is the most widely available, recommended intervention in persons with skeletal muscle dysfunction^28^. This nomogram showed satisfied discrimination and calibration performance in predicting malnutrition in COPD patients. It provides clinicians with scientific guidance and convenient approaches to identify individuals at high risk of malnutrition, thus intervening properly to improve patients’ quality of life and long-term prognosis.

However, some limitations of this study should be mentioned. This retrospective and single-centered study leads to potential selection bias, and only internal verification is shown. Considering the lower prevalence of COPD in females, we only included male participants. The results were incomplete since men's body composition differed from women's, especially in menopausal women. Future studies with larger sample sizes in multiple centers and longer follow-up periods should be conducted to confirm the accuracy of the currently observed relevant indicators. Recent researches have focused on the oxidative stress biomarkers and revealed that advanced oxidation protein products^17^, total radical trapping antioxidant parameter^29^ and resistin^30^ were signicant biomarkers related to sarcopenia in COPD. More serological markers need to be included and confirmed by further research.

## Conclusion

This study demonstrated the nutritional status and functional exercise capacity of patients with stable COPD. As the disease worsens, the proportion of malnutrition increases and the muscle shows a declining trend. To identify malnutrition early and convenient, the study developed a prediction score system to distinguish people at high risk of malnutrition and conduct rehabilitation program. The model has good internal validity and high discrimination, providing great clinical value for potential promotion.

## Data Availability

The data that support the findings of this study are available from the corresponding author upon reasonable request.
